# Association Between Commute Duration and Sickness Absence in the Context of China: Mechanism and Heterogeneous Effects

**DOI:** 10.3389/fpubh.2021.611162

**Published:** 2021-03-05

**Authors:** Zicheng Wang, Qiushi Wu, Murong Guo

**Affiliations:** ^1^School of Public Administration and Emergency Management, Jinan University, Guangzhou, China; ^2^School of Labor and Human Resources, Renmin University of China, Beijing, China

**Keywords:** commute duration, sickness absence, mechanism, health-related outcomes, work effort, heterogeneous effects

## Abstract

**Background:** Most employees in urban China have experienced a heavy commuting burden, which has become an urgent issue that should be solved in the new urbanization strategy process. However, the exploration of the relationship between the commute duration and sickness absence remains scant in China, and no direct discussion has been done to analyze the mechanism linking commute duration and sickness absence.

**Methods:** Using a unique dataset of the 2013 China Matched Employer–Employee Survey, the present study applies a two-level random-intercept Poisson model to explore this association.

**Results:** A long commute is significantly related with increased sickness absence. A longer commute is associated with poorer self-rated health status and a higher degree of psychological depression, and it is also highly related with a decrease in sleeping time. Moreover, an increased commuting duration is associated with lower work effort (working hours).

**Conclusion:** Longer commute duration induces lower productivity through increased sickness absence, and the potential link of commute duration and sickness absence is mainly transmitted through health-related outcomes and work effort.

## Background

Commuting is an indispensable part of daily life for millions of people worldwide (1). A large body of previous studies focused on the relationship between commuting and employees' labor market performance (2–4). The evidence shows that commuting has a negative effect on productivity in that increased commute duration is associated with increasing absence of workers due to sickness (2, 5).

Two potential mechanisms link the relationship between commute duration and workers' absenteeism. First, involuntary behaviors induce absence. From the new welfare economics theory, long commute duration is viewed as a time-consuming activity, which is related with poor psychological and physical health outcomes (6–8). Employees with long commute duration are more likely to suffer illness due to heavy stress and fatigue. Accordingly, increasing commute duration is associated with fewer health-promoting behaviors, such as physical activities and social participation (9). A long commute duration also would crowd out sleeping time and therefore induce negative health outcomes (2). Thus, employees with a long commute duration have more probability to suffer poor health outcomes and are therefore more prone to be absent for sickness than short commuters (10). The second mechanism emphasizes voluntary behaviors based on the urban efficiency wage model, which argues that workers' shirking behaviors (e.g., reducing work effort) and long commute duration are substitutes. Long commute duration would affect the time available for leisure at home, and therefore, employees would make a trade-off between commute duration and effort at work (11). Long commuters would experience a heavy monetary and non-monetary commuting cost, and then absence is considered to compensate for the commute cost. Therefore, a long commute would crowd out employees' available time for leisure at home, leading to shirking behaviors, and then increase the likelihood of sick leave (5).

Longer commute duration may induce more sickness absence. A body of evidence from developed markets has confirmed that longer commute duration might increase the likelihood of illness-related absence (2, 5, 12, 13), but several studies also reveal no evidence supporting this relationship (5, 14). More importantly, some studies provide evidence of heterogeneous effects. Using data from the Panel Study of Income Dynamics for 2011, 2013, and 2015, Gimenez-Nadal et al. (15) found that the daily commute is associated with men's sick-day absences, whereas it has no significant effect on those of women. Similarly, Karlström and Isacsson (16) pointed out that commute duration only has a positive effect on sickness absence of women with low wages.

Additionally, several studies from non-western countries also show clear evidence that long commute duration is linked with poor health status. Using a large and unique nationally representative sample in Brazil, Oliveira et al. (17) concluded that individuals with more than 1 h of commuting are more likely to be in poor health. Applying the Seoul survey data collected between 2006 and 2015, Jun et al. (18) also revealed a negative relationship between commute duration and subjective well-being. A survey conducted in Tokyo for school teachers by Nomoto et al. (19) also demonstrated that long-time commuters are more likely to have less sleep and exercise.

With the rapid urbanization and increasing ownership of private vehicles, most employees in urban China have experienced a heavy commuting burden (8). An increasing amount of researches has focused on the nexus between commuting and urban residents' subjective well-being (8, 20–22). However, discussions on the relationship between commute duration and sickness absence remain scant in China and should be further explored in the future.

In sum, previous studies have two limitations, as follows. First, most studies were carried out in European developed markets, whereas the discussion in the non-western or undeveloped context remains unexplored. Another limitation is that the mechanism linking commute duration and sickness remains unclear, and the debate on whether this relationship is transmitted through health-related outcomes or shirking behaviors is also underexplored.

To fill these gaps, this study attempts to address two issues: Is commute duration positively related with sickness absence? If it is, what is the potential mechanism linking commute duration and sickness absence?

This study may contribute to existing studies in several distinct ways.

First, following Goerke and Lorenz (5), a unique dataset, namely, China's Matched Employee–Employee Survey (CMEES), and the multilevel model are applied to explore the nexus between commute duration and sickness absence in the Chinese context.

Second, the two potential mechanisms linking commute duration and sickness absence are discussed, providing a new exploration in the Chinese context.

Third, this study provides new evidence on the relationship between commute duration and sickness absence in a group-specific estimation, showing that increasing commute duration is positively related with urban residents' sickness absence but has no significant impact on rural migrants' absenteeism. Meanwhile, active commuting shows an insignificant effect on sickness absence.

## Materials and Methods

### Data Source

We used the data from CMEES, which was conducted by the School of Labor and Human Resources, Renmin University. Commute duration was only available in the wave of 2013. CMEES-2013 covers 12 cities chosen from the eastern, middle, western, and northeastern regions of China, namely, Beijing, Guangzhou, Fuzhou, Jinan, Chengdu, Changchun, Taiyuan, Zhengzhou, Qiqihar, Xianyang, Suzhou, and Xiangyang. A total of 4,532 employees from 444 enterprises were covered in CMEES-2013. The distribution of enterprises surveyed across units ranges from 25 to 50, including 3 sampling units, namely, 25, 40, and 50. A total of 25 firms were sampled from prefecture-level cities, namely, Qiqihar, Xianyang, Suzhou, and Xiangyang, and 50 enterprises were sampled from Beijing and Guangzhou.

Those samples were drawn using the two-stage method of stratified sampling. The dataset was selected from an enterprise listing set up on the basis of the 2008 National Economic Census data. The sample was collected from managers who were responsible for employment relations or personnel matters in private and public sector companies with 20 or more staffs. If a sampled enterprise refused to respond, it was replaced by another company with the same firm size in the same industry.

CMEES-2013 not only collects rich information on the characteristics of company-level, demographic, and employment traits for employees but also provides detailed information about days absent for sickness and commute duration, facilitating the discussion on the association between commuting and sickness absence.

### Explanatory and Outcome Variables

The outcome variable is the annual number of days absent from work due to sickness, which derives from the following item in the survey: “In the past year, how many days have you asked for leave due to illness?”

The focal variable is the commute duration, which is described as minutes spent in one-way daily commute.

The control variables were chosen according to Goerke and Lorenz (5) as well as Gimenez-Nadal et al. (15), which are divided into two sets: personal characteristics and job-related traits.

Personal characteristics consist of age, male (ref=female), educational achievement (years completed), and migration status (ref=non-migrant). Job-related traits include occupation categories (ref=ordinary worker), job strain (a five-point Likert-type scale), overtime (hours per week), training (days per year), job tenure (years), job security (a five-point Likert-type scale), injury (ref=has no work-related injury during the past year) and wage (natural logarithm), company type (ref=domestic private enterprise), and industry (ref=other non-manufacturing industry).

In the mechanism analysis, as for the health-related status, subjective health indexes (self-rated health status and degree of depression, both of them measured by a five-point Likert-type scale) and objective health indicators (sleeping time, measured by sleeping hours per day) are viewed as outcomes. Working hours per week was set as a proxy for work effort to check the potential mechanism of shirking behaviors.

### Econometric Model

The number of days absent for sickness is a count variable (0, 1, 2, 3, and so on); the count responses Poisson model may be appropriate for this study. Following Goerke and Lorenz (5) and Gimenez-Nadal et al. (15), the determinants of sickness absence are mainly derived from an individual level, including personal characteristics and job-related aspects. Residence location is regarded as an important determinant for the length of the commute and the worker's absenteeism (2), meanwhile urban planning, public infrastructure, and built environment vary in different cities, which would lead to differentiated outcomes of jobs–housing balance (23); therefore, the city-level between-group variations of worker's absenteeism derived from the commute may be more substantial. Thus, we consider a two-level random-intercept Poisson model for discussion.

Level 1 is defined as the lowest level (individual level), and level 2 is viewed as the higher level (city level):

(1) $$\begin{array}{l} level\;1:\log\left(sickness\text{\_}a_{ij}\right)=\beta_{0j}+{\beta X}_{ij} \end{array}$$

(2) $$\begin{array}{l} level\;2:\beta_{0j}=\gamma_{00}+u_{0j} \end{array}$$

$$\begin{array}{l} Mixed\;Model:\log\left(sickness\text{\_}a_{ij}\right)=\gamma_{00}+{\beta X}_{ij}+u_{0j}\\ with\;1=1,2,.....N\left(Individual\right),j=1,2,....12\left(Cities\right) \end{array}$$

where sickness_*a*_*ij*_ represents the sickness absence days of a person i in a city j, *X*_*ij*_ is the control, γ_00_ is the random intercept, and *u*_0*j*_ is the dependence error.

## Results

### Descriptive Statistics

Table 1 shows the descriptive statistics. The average number of days absent due to sickness is nearly 2.42, whereas approximately 60% of employees have not been absent for illness, and only 1.9% of them have been absent for over 30 days. The mean one-way commute duration is approximately 26 min, and the maximum value reaches 120 min. Most urban workers in China suffer a heavy commute burden, with 22.4% of commuters with a daily commute over 39 min.

**Table 1:** Descriptive statistics.

**Variables**	**Mean**	**Std. dev**.	**Min**	**Max**
**Dependent variable**				
Sickness absence	2.4225	5.6750	0	60
Sickness absence dummy	0.5985	0.4903	0	1
**Independent variables**				
Commuting time (CT)	26.198	20.133	0	120
Long commuter (CT > 39)	0.2240	0.4169	0	1
**Control variables**				
Age	33.224	9.8471	16	72
Male	0.4545	0.4980	0	1
Married	0.6652	0.4720	0	1
Educational achievement	13.237	2.8078	6	19
Manager	0.2174	0.4125	0	1
Skilled worker	0.1771	0.3818	0	1
Ordinary worker	0.2050	0.4037	0	1
Job strain	2.9101	1.1178	1	5
Overtime (hours per week)	3.3669	5.3424	0	32
Training time (days per year)	6.8506	15.070	0	120
Job tenure (years)	5.3188	6.4301	0.5000	51
Job security	3.6180	0.7765	1	5
Injury	0.02339	0.1512	0	1
Wage (natural logarithm)	10.338	0.4957	9.0938	12.429
State-Owned enterprise (SOE)	0.1470	0.3541	0	1
Foreign-Owned enterprise (FOE)	0.05841	0.2346	0	1
Domestic private enterprise (DFE)	0.7928	0.4054	0	1
Manufacturing	0.3143	0.4643	0	1

### Benchmark Results

As shown in Table 2, the estimations from the baseline, Model (1), demonstrate that commute duration is positively related with sickness absence. The controls are also as expected. Sickness absence would increase with job strain, length of overtime, and monthly wage; absence would decrease with age, educational achievement, job tenure, and job security. Furthermore, men, unmarried workers, migrants, and ordinary workers are less likely to be absent due to sickness; employees in state-owned enterprises have a higher likelihood of experiencing sickness absence.

**Table 2:** Estimation of the relation between commuting time and sickness absence.

**Variable**	**Model (1)** **full sample**			**Model (2)** **sickness leave as dummy variable**			**Model (3)** **commuting as categorical variable (commute duration > 39)**			**Model (4)** **excluding Injury = 1**			**Model (5)** **added transport modes variables**		
	**IRR**	**SE**	***P* > |z|**	**OR**	**SE**	***P* > |z|**	**IRR**	**SE**	***P* > |z|**	**IRR**	**SE**	***P* > |z|**	**IRR**	**SE**	***P* > |z|**
CT	1.004	0.001	0.000	0.004	0.002	0.047	1.163	0.031	0.000	1.004	0.001	0.000	1.003	0.001	0.000
Age	0.986	0.002	0.000	−0.009	0.005	0.071	0.986	0.002	0.000	0.984	0.002	0.000	0.986	0.002	0.000
Male	0.734	0.017	0.000	−0.413	0.074	0.000	0.731	0.017	0.000	0.748	0.017	0.000	0.732	0.017	0.000
Married	1.117	0.032	0.000	−0.128	0.093	0.167	1.117	0.032	0.000	1.141	0.033	0.000	1.106	0.032	0.000
Educational achievement	0.957	0.004	0.000	−0.012	0.015	0.449	0.959	0.004	0.000	0.960	0.005	0.000	0.957	0.004	0.000
Migrant	0.824	0.025	0.000	−0.087	0.093	0.347	0.817	0.024	0.000	0.833	0.026	0.000	0.836	0.025	0.000
Manager	1.034	0.031	0.276	−0.064	0.099	0.519	1.031	0.031	0.316	1.019	0.032	0.541	1.032	0.031	0.297
Skilled worker	1.142	0.032	0.000	0.095	0.091	0.299	1.139	0.032	0.000	1.133	0.032	0.000	1.146	0.032	0.000
Job strain	1.076	0.010	0.000	0.114	0.032	0.000	1.077	0.010	0.000	1.093	0.011	0.000	1.076	0.010	0.000
Overtime	1.008	0.002	0.000	0.029	0.007	0.000	1.009	0.002	0.000	1.010	0.002	0.000	1.009	0.002	0.000
Training	1.000	0.001	0.923	−0.005	0.002	0.062	1.000	0.001	0.810	1.000	0.001	0.682	1.000	0.001	0.995
Job tenure	0.994	0.002	0.003	−0.012	0.007	0.091	0.993	0.002	0.002	0.994	0.002	0.005	0.994	0.002	0.003
Job security	0.952	0.013	0.000	−0.099	0.045	0.029	0.952	0.013	0.000	0.938	0.013	0.000	0.949	0.013	0.000
Injury	1.776	0.095	0.000	0.133	0.223	0.549	1.758	0.094	0.000	–	–	–	1.794	0.096	0.000
Log (wage)	1.022	0.028	0.439	0.234	0.088	0.008	1.026	0.028	0.359	1.001	0.028	0.980	1.010	0.028	0.708
SOE	1.024	0.033	0.467	−0.226	0.108	0.037	1.022	0.033	0.503	0.992	0.033	0.799	1.023	0.033	0.483
FOE	0.840	0.043	0.001	−0.134	0.154	0.384	0.838	0.043	0.001	0.844	0.045	0.001	0.838	0.043	0.001
Manufacturing	1.129	0.026	0.000	0.332	0.077	0.000	1.127	0.026	0.000	1.151	0.027	0.000	1.131	0.026	0.000
Active_mode	–	–	–	–	–	–	–	–	–	–	–	–	0.893	0.023	0.000
Constant	4.890	1.405	0.000	−2.203	0.899	0.014	4.851	1.393	0.000	5.982	1.760	0.000	5.809	1.689	0.000
city var (_cons)	0.060	0.025		0.177	0.080		0.060	0.025		0.067	0.028		0.064	0.027	
Log likelihood	−14,739.40			−2,479.34			−14,748.03			−14,032.95			−14,729.44		
*N*	3,839			3,839			3,839			3,746			3,839		

*(1) Model: multilevel mixed-effects Poisson regression is used in all models except for Model (2) (multilevel mixed-effects logistic regression). (2) The likelihood-ratio test is highly significant in all models*.

Several group-specific models were performed to verify the sensitivity of the main findings, as shown in Table 2, Models (2–5). To correct the measurement error, we also defined sickness absence as a dummy variable (it equals 1 if the individual took sick leave during the past year) to provide additional analysis of the commute–absenteeism relationship in Model (2). Similarly, a long commute duration may be related to higher levels of fatigue (15), inducing heavy sickness absence. Thus, we divided commuters into two subgroups to estimate in Model (3), where the commuters with more than 39 min commute duration are defined as long commuters^1^. Moreover, the commuters who experienced injuries at work are more likely to obtain sickness absence permission. Model (4) is used to re-estimate for those who have not been injured at work during the past year. Active travel commuters are more likely to be healthy (23), thereby decreasing sickness absence. In Model (5), the variables of the transportation modes are incorporated. The active mode refers to those who walked or cycled to work, and the passive mode includes those who drove cars or used public transportation.

As shown in the estimations in those models, a robust positive association occurs between commute and sickness absence against several specifications.

### Mechanism Analysis

Two possible mechanisms link the commute and sickness absence. One is that a long commute might weaken employees' health status, inducing involuntary or unavoidable absenteeism. Another is that commuting may induce shirking behaviors, increasing the probability of voluntary or avoidable absenteeism. Health-related status, such as subjective health indexes (self-rated health status, degree of depression) and objective health indicators (sleeping time) are incorporated as outcomes in Models (6–8). With the dataset unavailable, we applied working hours per week as a proxy for work effort to check the potential mechanism of shirking behaviors in Model (9).

As shown in Table 3, longer commute duration is associated with poorer self-rated health status and a higher degree of psychological depression, and it is also highly related with decreased sleeping time. Results also reveal that commute duration is negatively associated with working hours. The estimations in Table 4 also show that good health outcomes are negatively related with high sickness absence; that is, increased sickness absence is linked with poor self-rated health status, low sleeping time, and high depression. The results also demonstrate that increased working hours are related with increasing sickness absent days.

**Table 3:** Mechanism analysis: health outcomes and work effort.

**Variable**	**Model (6) self-rated health**			**Model (7) depression**			**Model (8) sleeping time**			**Model (9) working time**		
	**OR**	**SE**	***P* > |z|**	**OR**	**SE**	***P* > |z|**	**β**	**SE**	***P* > |z|**	**β**	**SE**	***P* > |z|**
CT	0.9953	0.002	0.009	1.005	0.002	0.034	−0.025	0.007	0.000	−0.0344	0.0073	0.000
Constant	N/A	N/A	N/A	N/A	N/A	N/A	54.34	3.014	0.000	68.02	3.247	0.000
city var (_cons)	0.1179	0.054		0.1181	0.0563		6.911	1.087		2.538	0.4557	
Log likelihood	−3,578.04			−4,460.0			−13,114.6			−4,460.0		
*N*	3,839			3,836			3,789			3,836		

*(1) Models (6) and (7) are used in multilevel mixed-effects ordered logistic regression; Model (8) and Model (9) are used in a multilevel mixed-effects generalized linear model. (2) The likelihood-ratio test is highly significant in all models. (3) Like Table 2, all control variables are included*.

**Table 4:** Association between sickness absence and health outcomes or work effort.

**Variable**	**Model (6_a) self-rated health**			**Model (7_a) depression**			**Model (8_a) sleeping time**			**Model (9_a) working time**		
	**OR**	**SE**	***P* > |z|**	**OR**	**SE**	***P* > |z|**	**OR**	**SE**	***P* > |z|**	**OR**	**SE**	***P* > |z|**
Self-rated health	0.7413	0.116	0.000									
Depression				1.278	0.0532	0.000						
Sleeping time							0.9049	0.0197	0.000			
Working hours										1.0099	0.0011	0.000
Constant	7.122	0.4290	0.000	0.3821	0.0583	0.000	2.627	0.0346	0.000	1.6028	0.0846	0.000
city var (_cons)	0.0330	0.0030		0.1489	0.0682		0.0627	0.0059		0.0465	0.0057	
Log likelihood	−14,916.2			−2,531.4			−15,079.1			−15,009.6		
*N*	3,839			3,836			3,839			3,824		

*(1) Model: Model (6_a), Model (7_a), Model (8_a), and Model (9_a) are used in the multilevel mixed-effects generalized Poisson model. (2) The likelihood-ratio test is highly significant in all models. (3) Like Table 2, all control variables are included*.

### Heterogeneous Effects

In this section, we attempt to estimate the heterogeneous effects of commute duration on absenteeism for sickness with respect to *hukou* status, gender, and commuting mode. As for the *hukou* system, we split the sample into rural migrant and urban resident groups. Individuals with a non-local agricultural *hukou* are defined as rural migrants; otherwise, employees with local urban *hukou* belong to the groups of urban residents. The commuting modes are divided into active and passive ones.

The estimations as shown in Table 5 indicate that commute duration is positively associated with urban citizens' sickness absence but has no significant effect on rural migrants. Similarly, a commute only has a positive influence on men's absence, but it has no significant influence on women. With regard to the transportation mode, no significant evidence was found in either active or positive groups.

**Table 5:** Heterogeneous effects with respect to *hukou* status, gender, and transportation modes.

	***hukou*** **system**						**gender**						**transportation modes**					
**Variables**	**Model (10a)** **Migrant = 1**			**Model (10b)** **Migrant = 0**			**Model (11a)** **Male = 1**			**Model (11b)** **Male = 0**			**Model (12a)** **Active mode = 1**			**Model (12b)** **Active mode = 0**		
	**IRR**	**SE**	***P* > |z|**	**IRR**	**SE**	***P* > |z|**	**IRR**	**SE**	***P* > |z|**	**IRR**	**SE**	***P* > |z|**	**IRR**	**SE**	***P* > |z|**	**IRR**	**SE**	***P* > |z|**
CT	0.999	0.001	0.333	1.004	0.001	0.000	1.002	0.001	0.032	1.003	0.002	0.191	1.005	0.007	0.440	1.002	0.002	0.224
Constant	70.465	50.901	0.000	3.287	1.021	0.000	7.632	3.412	0.000	0.045	0.057	0.013	1.449	2.711	0.843	1.429	0.042	0.079
City var (_cons)	0.308	0.134		0.048	0.020		0.076	0.033		0.296	0.139		0.134	0.083		0.057	0.034	
Log likelihood	−2,504.7			−12,034.36			−6,115.38			−1,353.68			−2,125.5			−4,623.2		
*N*	796			3,043			1,747			2,092			1,273			2,566		

*(1) Model: multilevel mixed-effects Poisson regression is used in all models. (2) The likelihood-ratio test is highly significant in all models. (3) Like Table 2, all control variables are included*.

## Discussion

The benchmark results demonstrate that employees might be inclined to ask for illness-related absences with increases in commute duration, and this is consistent with the findings by Van Ommeren and Gutiérrez-i-Puigarnau (2) and Goerke and Lorenz (5), which also confirmed a positive nexus between commute duration and sickness absence. This result is still robust against several specifications.

The results from the mechanism analysis reveal that longer commuting duration is associated with poorer subjective (self-rated health and depression) and objective (sleeping time) health status. This finding is consistent with previous evidence that commuting has been linked to negative health-related outcomes (8, 9, 24). In this scenario, health-related outcomes do act as an important transmission channel linking the relationship between commuting and sickness absence. This finding is in line with Gimenez-Nadal et al. (14), which also depicts the association between commuting and workers' health-related outcomes. This finding implies that more time spent on commuting might break the work–life balance among employees and tend to push more burdens on both objective and subjective health status, including a combination of factors such as tension, tiredness, depression, irregular diet, and low sleeping time, possibly leading to the greater likelihood of involuntary or unavoidable sickness absenteeism and lowering productivity.

The mechanism analysis also demonstrates that increasing commute duration is negatively related with actual weekly work time, implying that commuting duration will increase the probability of shirking behaviors. This finding is consistent with Ma and Ye (23), who pointed out that commute distance is negatively related with work participation and engagement. This evidence implies that the employees who experience heavy commuting burdens are more likely to reduce their work effort.

The estimations of heterogeneous effects show interesting results. Commute duration is related with urban residents' sickness absence, but it has no significant effect on rural migrants. These findings are in contrast with the finding by Chia (25), who suggested that migrants in Singapore have a higher possibility of sickness absence than their local counterparts. The potential explanation is that rural migrants' access to public health services is legally restricted by the *hukou* system (26). Once they get sick, they need to choose a private clinic nearby rather than a formal hospital to obtain medical service (27). These unregulated private clinics usually fail to provide official certificates for migrants to obtain sick leave permission. Without sick leave permission, these rural migrants may suffer an extra economic loss of days off work, so they are less likely to be absent even if they are ill or uncomfortable (28).

A commute is positively related with men's absence, but it has no significant influence on women's absence. This finding is consistent with Gimenez-Nadal et al. (15), who draw the same conclusion in the United States. This gender differential may be due to women's shorter commute times. Gender stratification remains a serious problem in China, in which women often bear disproportionately heavy household responsibilities, including housework and childcare (29–31).

For the active modes, commute duration has a positive but no significant influence on sickness absence. This finding is different from those of previous studies, which confirmed that active commuting is related with better health status and less sickness absence (32–34). The potential reason may be that cycling in China may induce worse health status, which is different from other developed countries because non-motor vehicle traffic plans and availability of public bicycle facilities are ignored by the local government (35). Thus, insufficient bicycle lanes make cyclists more vulnerable in mixed traffic, which may threaten their health through potential bike–automobile collisions (36).

## Conclusion

### Main Findings

With the rapid urbanization in China, ensuring work–life balance and improving health status for employees have been an urgent issue in occupational health security. This study confirms that commuting duration is positively related with sickness absence, whereas it remains robust against several specifications. This study also points out that health-related outcomes and work effort for employees mainly act as a mechanism. Additionally, the heterogenous effects of commutes on absenteeism for sickness are differentiated across *hukou* status and gender. These findings demonstrate that commute duration is positively related with urban residents' sickness absence but has no significant relationship with that of rural migrants. Commute duration is also positively related with men's absence but has an insignificant influence on that of women. Otherwise, passive and active commuting modes show insignificant effects on sickness absence.

### Implications

This study has several implications.

First, long commuting duration may reduce workers' productivity through high sickness absence. Thus, employers should reduce the commuting burden of their workers. A daily shuttle service from employees' residences to workplaces should be provided firstly to reduce the commuting burden. Then, employers could adopt more flexible work schedules to alleviate the work–life conflict. For example, the staggered rush hour plan should be adopted widely, which allow the employees choose a flexible working time from 8 a.m, 9 a.m, or 9:30 a.m to reduce the commute duration and disperse peak hours. Moreover, a commute allowance or commercial health insurance should be enhanced to help long commuters to recover.

Second, policymakers should improve transportation networks and services to provide faster and shorter commuting. Improving the jobs–housing balance must be prioritized in the process of new urbanization to relieve the heavy burden. Urban planning should attach great importance to the jobs–housing balance, including increasing residential opportunities in business districts or increasing employment opportunities in residential districts.

Moreover, the local government should increase financial investment in improving public transportation systems; bus rapid transit, suburban railways, and subways should be developed on a large scale in metropolitan areas.

### Limitations

This study also has certain limitations. First, the applied dataset of CMESS-2013 is cross-sectional data. The potential endogeneity might need to be addressed to explore the causal link between commutes and absenteeism for sickness in further studies. Second, unobserved factors, especially poor health status or specific illness, should be addressed in the future. Third, the mechanism of shirking behavior is under-discussed. The relationships between commute duration and work effort should be addressed under a specific measurement of work effort.

## Data Availability Statement

The data analyzed in this study is subject to the following licenses/restrictions: The CMEES that support the findings of this study are available from School of Labor and Human Resources, Renmin University, but restrictions are applied to the availability of these data, which were used under license for the current study, and so are not publicly available. CMEES are however available from the authors upon reasonable request and with permission of School of Labor and Human Resources, Renmin University. China's Matched Employer-Employee Survey Data Access can be contacted for more information (yuhui_li@ruc.edu.cn; df594133@163.com). Requests to access these datasets should be directed to yuhui_li@ruc.edu.cn; df594133@163.com.

## Ethics Statement

This study is a secondary analysis based on the data from the CMEES conducted by School of Labor and Human Resources, Renmin University, all of which were subject to multiple stages of reviews by experts to address methodological, ethical, and legal issues related to data collection. Final approvals of all CMEES surveys were required from the Research Ethics Committee of Renmin University to ensure that the data collection complied with ethical requirement according to the Statistics Act.

## Author Contributions

ZW took leadership and responsibility for the research activity planning and made substantial contributions to the conception and design of the Programme. QW worked on the statistical analysis of the data. MG drafted the concept of the paper as well as participated in finalizing the manuscript. All authors read and approved the final manuscript.

## Conflict of Interest

The authors declare that the research was conducted in the absence of any commercial or financial relationships that could be construed as a potential conflict of interest.

## Footnotes

^1^We choose 39 min as the boundary between long and short commuters because it is the average commuting duration from the 2016 report from the National Commuting Survey in China.
